
JOURNAL INFORMATION
==============================
NLM Title Abbreviation: ScientificWorldJournal
Journal ID: ScientificWorldJournal
NLM Journal ID: 101131163
Journal ID: TSWJ

The Scientific World Journal

ISSN: 2356-6140
EISSN: 1537-744X
Publisher: Wiley

ARTICLE INFORMATION
==============================
PMCID: PMC5848795
PMID: 18454262
DOI: 10.1100/tsw.2008.77
Article ID: 587879
Article version: 1
Subjects: Clinical Image

Dramatic Verrucae Confined to the Lower Lip

Namazi M. R. 1 2 *
Jorizzo J. L. 1
1 Dermatology Department, Wake Forest University Health Sciences, Winston-Salem, North Carolina, USA
2 Shiraz University of Medical Sciences, Shiraz, Iran
*M. R. Namazi: namazi_mr@yahoo.comAcademic Editor: Daniel Batlle

Publication date: 2008
Electronic publication date: 2008 May 1
Volume: 8
First page: 507
Last page: 507
Received 2008 Feb 19; Accepted 2008 Apr 24
Copyright: Copyright © 2008 M. R. Namazi and J. L. Jorizzo.
Copyright year: 2008
License: This is an open access article distributed under the Creative Commons Attribution License, which permits unrestricted use, distribution, and reproduction in any medium, provided the original work is properly cited.
License URL: https://creativecommons.org/licenses/by/3.0/

Keywords: HPV, Wart, Verrucae

==============================
Clinical Image
*TheScientificWorldJOURNAL* (2008) 8, 507
ISSN 1537-744X; DOI 10.1100/tsw.2008.77

TheScientificWorldJOURNAL
www.thescientificworld.com

### Dramatic Verrucae Confined to the Lower Lip

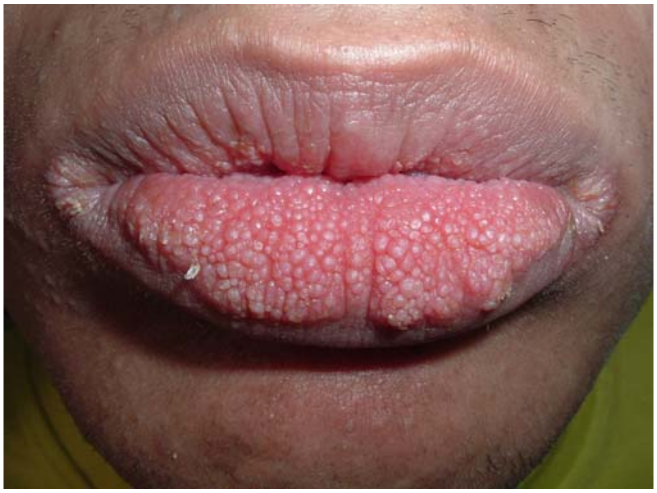

**FIGURE**. PCR for Human Papilloma Virus was not available.

M.R. Namazi[1,2,]* and J.L. Jorizzo[1]

*[1]Dermatology Department, Wake Forest University Health Sciences, Winston-Salem, North Carolina; [2]Shiraz University of Medical Sciences, Shiraz, Iran*

**E-mail:** namazi_mr@yahoo.com

*Received February 19, 2008; Accepted April 24, 2008; Published May 1, 2008*

**KEYWORDS**: HPV, Wart, Verrucae

A 16-year-old boy presented with a 3-year history of development of small papules on his lower lip. Histologic study of a biopsy specimen confirmed that the lesions were verrucae vulgaris. Other than having several genital warts, the patient was otherwise healthy and his HIV serology and full blood count were within normal limits.

The incubation period for common warts is 1–6 months. The causative virus most easily enters the body through an area of skin that is moist, peeling, or cracked. These lip lesions are particularly dramatic and unusual.

**This article should be cited as follows:**

Namazi, M.R. and Jorizzo, J.L. (2008) Dramatic verrucae confined to the lower lip. *TheScientificWorldJOURNAL* **8**, 507. DOI 10.1100/tsw.2008.77.

*Corresponding author.
©2008 with author.
Published by TheScientificWorld; www.thescientificworld.com
